# Evaluating the impact of light quality on plant–herbivore interactions using hemp as the model system

**DOI:** 10.1093/ee/nvad127

**Published:** 2024-01-10

**Authors:** Eze Pojmann-Ezeonyilo, Petrus Langenhoven, Laura L Ingwell

**Affiliations:** Department of Entomology, Purdue University, West Lafayette, IN, USA; Department of Horticulture and Landscape Architecture, Purdue University, West Lafayette, IN, USA; Department of Entomology, Purdue University, West Lafayette, IN, USA

**Keywords:** integrated pest management (IPM), indoor production, controlled environment

## Abstract

Light-emitting diodes (LED) offer energy-efficient and customizable light sources that can be tailored to optimize plant chemistry and growth characteristics. Indoor cannabis production is the most energy-intensive crop in the United States and suffers from insect pest infestations including the cannabis aphid, *Phorodon cannabis* Passerini, which can negatively impact yield. Here we investigated the potential of light quality (color) to manage *Cannabis sativa* plant chemistry and cannabis aphids to increase crop quality. *Cannabis* was grown indoors under LED lighting systems where we manipulated the color spectrum. Within each light treatment, a subset of plants was exposed to aphid herbivory. Physical and chemical plant responses and aphid biology were measured. The interaction between light quality and herbivory drove the time to the first flower (cola) in our experimental plants. Light quality did not impact THC/CBD, but plants under increased blue light had higher bud yield than those grown under white light. The red–blue light treatment resulted in the tallest plants with the lowest leaf-stem dry mass and bud yield. Herbivory decreased bud yield and lowered the concentration of CBD/THC in buds. Lastly, light quality impacted the reproduction and mortality of the cannabis aphid. This study demonstrates the capacity of light quality to impact plant growth traits but offers no evidence for light quality impacting CBD/THC production in *Cannabis*. More importantly, herbivory resulting from aphid feeding was shown to decrease CBD and THC. Light quality impacted pest biology, supporting the potential use of light quality as a pest management tool.

## Introduction

The cannabis aphid (*Phorodon cannabis* Passerini) is a pest and monophagous herbivore of *Cannabis* spp. (Cranshaw et al. 2018, Durak et al. 2021). The aphid feeds by piercing the plant with its mouthparts and tapping directly into the phloem, ingesting the sugary sap produced through photosynthesis. Aphid herbivory can adversely affect the fitness and yield of *Cannabis* plants through direct damage and the transmission of plant viruses and is exacerbated by their capacity for rapid population growth (McPartland et al. 2000, Durak et al. 2021, Pitt et al. 2022). Their high reproductive potential, in addition to their ability to transmit viruses, contributes to their pest status.

Herbivory can also affect plant chemistry through the induction of plant defenses (Stamp 2003). Damage from phloem-feeding insects, such as aphids, activates the salicylic-acid-mediated defensive pathways in plants, which increases the production of secondary metabolites that provide protection (Stamp 2003). In the case of *Cannabis*, terpenes and cannabinoids have been shown to have insecticidal properties against a variety of herbivores (Sirikantaramas et al. 2005, Górski et al. 2016, Park et al. 2019, 2022, Ahmed et al. 2020, Abendroth et al. 2023). CBD (cannabidiol) and THC (tetrahydrocannabinol) are just 2 of at least 85 cannabinoids found in *Cannabis sativa* (Hanuš et al. 2016) and are the most regulated chemicals in relation to hemp and marijuana production because of their medicinal and psychoactive properties. The floral buds of *C. sativa* are the most resinous part of the plant and, therefore, the structure where CBD and THC are most concentrated.

In controlled environment production, light is one of the most important (and expensive) components of the system. The advancements in lighting technology and increased availability of light-emitting diodes (LEDs) are revolutionizing the industry. In addition to reducing energy costs, LEDs offer the capacity to tailor light quality (color) to optimize production. Light quality has variable impacts on physical and chemical plant traits (Jeong, Chandra-Shekara, et al. 2010, Jeong, Kachroo, et al. 2010, Cerrudo et al. 2012, Cargnel et al. 2014, Moreno and Ballaré 2014). In fact, previous research has shown that light quality can be manipulated to alter the physical and chemical traits of *C. sativa.* Blue light regulates shoot and internode elongation, impacts leaf expansion and mass, increases flavonoids and polyphenols, and increases CBD content (Livadariu et al. 2018, Magagnini et al. 2018, Eichhorn Bilodeau et al. 2019). Red light increases yield and CBD content in bud tissues (Magagnini et al. 2018). Green light lowers THC levels (Mahlberg and Hemphill 1983, Magagnini et al. 2018), the tightly regulated psychoactive compound in this crop. Developing prescriptions of light quality shows promise as a tool to optimize crop production and regulate the production of CBD and THC. In markets where hemp is legal but marijuana is not, it is the concentration of these chemicals that delineates the crop as being compliant or noncompliant requiring crop destruction.

Light quality can have direct and indirect effects on plant–herbivore interactions. One direct effect that has been documented is the impact on the dispersion behaviors of the green peach aphid (*Myzus persicae* Sulzer), which are shown to be regulated by a phototaxic response to specific wavelengths (Zhang et al. 2016). Evidence of indirect effects includes UV-A radiation increasing the content of secondary metabolites benefiting the pest aphid but deterring the development of whiteflies in solanaceous crops (Dáder et al. 2014). Aphid fitness can also be reduced through light-mediated changes in the host plant’s chemical defenses (Rasool et al. 2020). Because the manipulation of light quality can regulate aspects of insect life history, and current evidence shows that it varies depending on the identity of individuals in the system, it has potential utility as an insect pest management tool in controlled environments.

Here we investigated how light quality can impact plant–herbivore interactions using hemp and the cannabis aphid as the model system. We designed our experiments to look at the direct effects of light quality on crop growth and cannabinoid chemistry, as well as the interactions between light quality and herbivory on crop production. Lastly, we measured the life-history traits of *P. cannabis* reared under different light-quality treatments to gain a better understanding of how light quality can impact plant–herbivore interactions for indoor hemp production. The work was conducted in a series of experiments carried out in indoor production spaces and growth chambers. We aimed to address the following questions: (i) How do light quality and herbivory interact to impact crop quality? (ii) Can light quality be used in an IPM plan to increase crop production?

## Materials and Methods

### Aphid Colony Maintenance

A colony of the cannabis aphid, *P. cannabis*, was maintained in the Department of Entomology at Purdue University and used as the source of aphids for this experiment. The colony originated from aphids collected from commercial hemp in Indiana in 2019. The species identification has been confirmed and voucher specimens added to the Purdue Entomological Research Collection (PERC). The aphids were reared in growth chambers (20.7 ± 0.9 C, 38.2% ± 2.8 RH, 20:4 L:D cycle; Percival Scientific, Perry, IA, USA) on ‘Little Giant’ autoflowering hemp plants (Old Country Hemp Co., NC, USA). The plants were replaced when they lost turgor.

### Light Quality Conditions

Hemp plants used in experiments were grown in an interior room in Smith Hall at Purdue University. LED light fixtures*—*Active Grow® 22W T5 4FT LED Strip Grow Lights (Seattle, WA, USA)*—*were the primary light source for the plants. Four light treatments were examined: (i) broad-spectrum white light, (ii) broad-spectrum white light during the day cycle supplemented by constant red light throughout the day and night, (iii) blue-heavy broad-spectrum lights, and (iv) a mixture of red and blue light. These treatments will henceforth be referred to as white, +red, +blue, and red–blue. The LED light fixtures were mounted onto 48” × 18” × 72” wire shelving units, with each light treatment being affixed to a separate shelf. In addition to the broad-spectrum Active Grow LED light from above, the +red plants received red light laterally from mounted LED lamps during both the day and night cycle (Westinghouse 33147 3314700 15W PAR38 Outdoor LED Red E26). Each shelf was covered with reflective biaxially oriented polyethylene terephthalate—aka BoPET or Mylar—to increase light intensity around the plants and prevent light from one treatment contaminating surrounding treatments. The photosynthetic photon flux density (PPFD) and light quality composition of each fixture was measured using a UPRtek (Zhunan Township, Taiwan) PG100N handheld spectrometer by averaging 3 readings taken directly under the fixtures from the height of the plant after germination and are reported in Table 1. A breakdown of the proportion of each color, by treatment, is shown in Supplementary Fig. S2. The lights were illuminated for a 20-h day interval. This photoperiod was determined to minimize shade stress while still having an adequate night period (4 h). Because the different colors of light fixtures produced similar PPFDs, the photoperiod was the same for all treatments, with the +red treatment being an exception. The daily light integral of each treatment was calculated, which quantifies the amount of photosynthetically active light in each area per day (Table 1). Each week, the plants were rearranged on the growing shelves following a clockwise rotation. This was done to control for variations in light intensity across the shelves.

**Table 1.** The PPFD (photosynthetic photon flux density) of the light sources was expressed as red, green, and blue wavelengths for each light treatment in the plant quality experiments. The total PPFD is the sum PFD (photon flux density) of red, green, and blue wavelengths. PPFD is measured here by μmol·m^−2^·s^−1^_._ The DLI (daily light integral) of each light treatment was measured in mol·m^−2^·day^−1^. DLI quantifies the amount of light available per day

	White treatment	+Red treatment (day)	+Red treatment (night)	+Blue treatment	Red–blue treatment
Red light PFD 600–700 nm (μmol·m^−2^·s^−1^)	106.1 ± 1.0	149.7 ± 12.9	16.8 ± 2.7	69.8 ± 2.6	184.2 ± 1.6
Green light PFD 500–600 nm (μmol·m^−2^·s^−1^)	89.3 ± 1.1	71.0 ± 7.1	0.2 ± <0.1	81.5 ± 2.6	13.6 ± 0.3
Blue light PFD 400–500 nm (μmol·m^−2^·s^−1^)	41.7 ± 0.5	43.5 ± 3.2	0.2 ± <0.1	87.4 ± 3.5	35.2 ± 1.0
Total PPFD (μmol·m^−2^·s^−1^)	235.5 ± 2.6	251.9 ± 3.3	17.2 ± 2.8	237.2 ± 9.3	232.1 ± 2.8
DLI (mol·m^−2^·day^−1^)	16.96 ± 0.19	18.39 ± 0.24		17.08 ± 0.36	16.71 ± 0.20

### Plant Material

The hemp variety ‘Little Giant’ was chosen for this experiment because it has a high CBD:THC ratio and, as an autoflowering variety, daylength did not need to be adjusted. The hemp seeds were sown directly into 2.26-liter pots and placed under the LED light fixtures on each rack. In each pot, one seed was sown into a 3:2 mixture of peat:perlite. The seeding depth was approximately 15 mm below the surface. Under each light treatment, 48 plants were grown.

Initially, the pots were bottom watered with tap water until the growing media was saturated. Subsequent irrigation was done with a watering can on the soil surface using a 180-ml fertilizer solution, alternating between a calcium nitrate solution made from Jack’s Nutrients 15-0-0 calcium nitrate (0.64 g/l solution) and Jack’s Nutrients 20-3-19 Petunia FeED Plus Mg (1.01 g/l solution) following the manufacturers’ recommended dilutions. The volume of the fertigation solution used per plant was increased to 800 ml on week 4. All plants received the same amount of water/fertilizer. The growth room had a mean day temperature of 23.2 °C ± 0.1 and a mean night temperature of 22.2 °C ± 0.1. The mean relative humidity was 40.7% ± 0.1 during the day period and 41.1% ± 0.3 at night.

The week the seeds were sown was denoted as week 1. The seeding date was recorded as well as the day the seedlings emerged and were completely freed from the pericarp and seedcoat. Seedlings that had sprouted but retained the pericarp and/or seedcoat were manually freed. The plants were observed weekly for the development of a cola, the dense cluster of floral buds at the terminal end of the hemp stem. Cola development is integral to the success of a CBD hemp crop because it is from the cola’s buds that CBD is extracted. For the terminus of a stem to be considered a cola in this study, the buds needed to be dense enough to obscure 2.5 cm of the stem.

### Cannabis Aphid Infestation

To test the effect of herbivory, half the plants across each light treatment were infested with 5 mature cannabis aphids on week 7 (96 plants total) and denoted as the “with aphids” treatment. The remaining 96 plants served as the “without aphids” treatment (herbivore-free control). From the aphid colony maintained in the laboratory and using a size 20 camel hair paintbrush, each aphid was transferred onto the experimental plant individually. Aphids were dislodged from their host plant by either tapping the plant to cause the aphids to naturally let go of the plant or by prodding their posterior end with the paintbrush to dislodge their mouth parts prior to moving them. Mature aphids were identified according to size and the presence of nymphs at the posterior location of the aphid on the host plant. All 5 aphids were confined to a single mature leaf on the infested plants using a cage made from a perforated bread bag. The bag was clamped shut using a wooden clothes pin and the inner lip of the bag and the leaf petiole was coated with petroleum jelly to impede the escape of aphids. When the leaflet began to yellow, the aphids and cages were secured to a new leaflet on the plant. In cases where the initial infestation of aphids did not survive, 3 new mature aphids were introduced to the cage.

### Tissue Sampling

Nine weeks after germination and 2 wk post-aphid infestation, samples of bud tissues were collected from each hemp plant to measure the content of CBD, CBDA, THC, and THCA. From each of the 8 treatment groups, 4 samples were collected. Each sample consisted of a pool of tissue collected from 4 randomly selected plants in the treatment. The top 25 mm of the apical cola of each of the 4 plants was cut off and constituted a single sample. The sample was flash-frozen inside a plastic vial and then stored in a −80 °C freezer. The bud tissue samples were delivered to Agrozen Laboratory (Lebanon, IN, USA). In brief, samples were oven-dried at 40 °C and then ground into a homogenous powder with a mortar and pestle. Cannabinoids were extracted using MeOH. Each fraction was analyzed using high-performance liquid chromatography (Agilent 1100 Series) equipped with a phenyl 5-µm column and coupled with variable wavelength detection. The flow rate was maintained at 1.5 ml/min with column temp at 50 °C. VWD was performed at 228 nm. Data were analyzed using ChemStation software (version B.04.03-SP). Identification was determined based on the retention time of CBD, CBDA, THC, and THCA and concentrations calculated from calibration curves using the 100 and 10 µg/ml standards for each analyte (Agrozen Labs detailed procedure). CBDA concentration was pooled into CBD and THCA concentration was pooled into THC.

### Aphid Population Management

To monitor the population growth of the aphids on the test plants, the whole plant was assessed on week 10 because the aphids had escaped the perforated bags after the tissue sampling. The scale used to estimate the number of aphids per plant included the following size bins: 0, ≤10, >10, >50, >100, >300, >500, >1,000. Aphid population densities were measured again on week 13, when the aphids had spread to both “with aphid” and “without aphid” treatments. A subset of 6 plants per light × herbivore treatment was assessed by counting the number of aphids on a center leaflet below the cola and originating from the upper third of the plant. These population measures are listed in Supplementary Table S1. On week 13, permethrin (Permethrin; Loveland Products, Inc; Greeley, CO, USA) was applied to the control plants at a rate of 1.0 gal/750 ft^2^ using a 1-gallon hand sprayer (Ace Home & Garden). Five days later, a second insecticide application was made using Beleaf 50 SG (a.i. flonicamid; FMC; Philadelphia, PA, USA) applied to the control ‘herbivore free’ plants at a rate of 0.063 oz/1,000 square feet using the same hand-pressurized sprayer.

### Hemp Biomass Harvest

On week 14, the hemp plants were harvested and dried. The experimental timeline is presented in Supplementary Fig. S1. The plants were severed at the surface of the growing medium. The colas were removed from the rest of the stem, as were any additional floral buds below the colas. The mass of the cola and buds were recorded as the bud wet mass. The remainder of the above-ground tissue was measured as leaf-stem wet mass.

The bud and leaf-stem tissues of each plant were stored separately in individual brown paper bags. The samples were oven-dried in batches due to limitations in the size of the oven. Each tissue batch spent at least 36 h in the drying oven at a temperature of 70 °C. To ensure the samples were thoroughly dehydrated, 3 bud samples and 3 leaf-stem tissue samples were removed from the oven, weighed, placed back in the oven for an additional 24 h, and then reweighed. If the second dry mass of the samples was within 10% of the first recorded dry mass, all the samples from the corresponding batch were considered adequately dried and weighed.

### Aphid Life History

This experiment was carried out in 2 CARON model 7312-22-1 plant growth chambers. White, +red, +blue, and red–blue light treatments were replicated within the growth chambers using the built-in LED light array. The 2 chambers were each divided into 2 shelf units (4 shelves total) with one light treatment replicated on each of the 4 shelves. The PPFD of each light treatment was calibrated by adjusting the brightness of the chamber’s blue, white, and red LED arrays, measuring red–blue–green ratios with a UPRtek PG100N handheld spectrometer and comparing with the ratio of the light treatments in the previous experiment (Table 2). The red–green–blue light compositions of the light treatments are shown in Supplementary Fig. S3. The interior of the growth chambers was lined with BoPET (Mylar) to maximize light dispersal. The chamber’s humidity was set to a constant 70%. The chambers underwent a daily cycle of 20:4 day:night with a daytime temperature set to 25 °C and a nighttime temperature set to 21 °C. The daytime PPFD of the chamber was set to 110 across each light treatment and the nighttime PPFD was set to zero.

**Table 2.** The PPFD (photosynthetic photon flux density) by light treatment, along with a breakdown of PFDs (photon flux densities) for each color category: red, green, and blue wavelengths for the growth chamber experiments evaluating aphid life history traits. Values were measured using a UPRtek PG100N handheld spectrometer (Zhunan Township, Taiwan) and rounded to a single decimal point. The DLI (daily light integral) of each light treatment, measured in mol·m^−2^·day^−1^, quantifies the amount of light available per day

	White treatment	+Red treatment (day)	+Red treatment (night)	+Blue treatment	Red–blue treatment
Red light PFD 600–700 nm (μmol·m^−2^·s^−1^)	68.7 ± 1.6	63.1 ± 1.3	4.0 ± 0.1	40.3 ± 0.7	104.1 ± 2.2
Green light PFD 500–600 nm (μmol·m^−2^·s^−1^)	35.6 ± 0.5	35.7 ± 1.5	0.1 ± < 0.1	39.7 ± 2.8	0.9 ± 0.1
Blue light PFD 400–500 nm (μmol·m^−2^·s^−1^)	19.6 ± 0.28	19.8 ± 0.9	< 0.1 ± <1.0	38.1 ± 0.9	17.6 ± 0.1
Total PPFD (μmol·m^−2^·s^−1^)	123.8 ± 2.3	118.6 ± 2.6	4.1 ± 0.1	118.1 ± 2.9	122.2 ± 2.1
DLI (mol·m^−2^·day^−1^)	8.80 ± 0.2	8.89 ± 0.2		8.91 ± 0.2	8.50 ± 0.2

Twenty-eight CBD hemp plants—*C. sativa* variety ‘Little Giant’—were grown from feminized seeds. Seven seeds were sown and grown under each of the 4 light treatments. The seeds were sown directly into 500 ml, circular, plastic pots in a 60:40 mix of peat and perlite. The plants were fertigated in unison when the substrate approached dryness with 300 ml of fertilizer solution, alternating between calcium nitrate solution made from Jack’s Nutrients 15-0-0 calcium nitrate (0.64 g/l solution) and Jack’s Nutrients 20-3-19 Petunia FeED Plus Mg (1.01 g/l solution) following the manufacturers’ recommended dilutions.

Four weeks after germination the hemp plants were infested with 2 mature, wingless aphids each (14 aphids per light treatment, 56 aphids in total). Each aphid was confined in a cage consisting of a 5-cm-long piece of 15.9-mm-diameter dialysis tubing (Thermo Fisher Scientific, Waltham, MA, USA) and sealed at each end with a foam stopper. The cages were fitted over a central leaflet of the hemp plant. When an aphid produced a nymph, the mother was removed, as were any extra nymphs. This sole remaining nymph in the cage was denoted as the “subject aphid” and was then monitored daily over the course of its life span.

The date the subject aphid was first observed was recorded as its date of birth and denoted as “Day 1” of its development. Daily, each exuvium the subject aphid produced was recorded and then removed using either tweezers or a paintbrush. When the subject aphid reached maturity and started producing its own offspring, the number of offspring was recorded daily, and each of the offspring was removed from the cage using tweezers or a paintbrush. The cause and date of each subject aphid’s death was recorded. In the cases where the aphids lived beyond their reproductive period, we recorded this and measured postreproductive length. Deaths were categorized as unnatural deaths—deaths caused by human error—and natural deaths. One of the most common causes of unnatural death was denoted as a “dry leaf death” (DLD). DLD occurred when circulation to the hemp leaflet was restricted by the aphid cage, causing desiccation of the leaflet and ultimately the death of the aphid. Attempts to avoid DLD were made by moving subject aphids to healthier leaflets when their current leaflet showed signs of desiccation or chlorosis. Other causes of unnatural deaths included being wounded or dropped during daily observations and maintenance of the subject aphid and its cage. Instances of subject aphids escaping were recorded as such and were categorized under deaths due to unnatural causes.

### Data Analysis

The plant–herbivore–light quality experiment was a full factorial design consisting of 4 light treatments—white, +red, +blue, and red–blue—which were crossed with the following 2 herbivore treatments: with aphids and without aphids (control). The main effects (herbivore treatment, light treatment) and their interactions were tested as predictive variables explaining differences in plant physical traits (height, time to first cola production, bud mass, leaf-stem mass) and chemical traits (% CBD, %THC, CBD:THC) using the fit model platform with a standard least squares personality in JMP Pro 16 (SAS Institute Inc, Cary, NC, 1989–2021). Plant growth and yield were compared on a per-plant basis (*n* = 192). Plant chemistry was examined by analytical sample (*n* = 32). A log transformation was performed on the leaf-stem dry mass data to normalize its distribution.

The aphid life-history experiment analyses focused on early life-history traits because they included more living individuals; many subject aphids died throughout the experiment—primarily due to unnatural causes. The developmental day on which molts occurred and the first offspring were produced were recorded and analyzed as a metric of development rate. The day of the aphid’s first molt (molt 1) was analyzed independently from the other molts because it has the highest number of aphids across treatments (*n* = 41). The effect of light treatment on the day of molt 1 was examined using nonparametric comparisons for each light treatment pair using the Wilcoxon/Kruskal–Wallis tests (ranked sums), and a 1-way chi-square approximation. The same analyses were used to examine the effect of light treatment on the aphid’s first day of reproduction (*n* = 33).

The analysis of fecundity was restricted to the first 6 days of each aphid’s reproductive period to preserve statistical power. Day 1 of the reproductive period was excluded to account for variation in the time between observations and the aphids true start of reproduction. By analyzing the 5-day period of days 2–6, the analyses included all but 2 aphids that reached reproductive maturity (*n* = 31). The effect of light treatment on the number of offspring produced by the subject aphid was analyzed using a standard ANOVA.

The interaction between light treatment and the rate of natural deaths over the course of development was analyzed to examine the effects of light quality on aphid mortality and longevity. To evaluate this, an aphid mortality index was calculated as follows:


$$\begin{array}{lll} y= & \overset{32}{\underset{x=1}{\sum }}\left\{\frac{\;Natural\;deaths\;on\;Day\;(x)}{Aphids\;alive\;on\;Day\;(x)}\right\}\; & +\left\{\frac{\;Natural\;deaths\;on\;Day\;(x+1)}{Aphids\;alive\;on\;Day\;(x+1)}\right\} \end{array}$$

This value measures the rate of natural deaths over time as a cumulative value in proportion to the constantly changing number of subject aphids in each light treatment. The values generated from this formula were plotted as a 1-way analysis with light treatment being the x-factor. The analysis is restricted to days 1–32 because the +blue light treatment had no surviving aphids at day 33. The significance of the interaction between light treatment and the aphid mortality index was analyzed using nonparametric comparisons for each light treatment pair using the Wilcoxon/Kruskal–Wallis tests (ranked sums) and a 1-way chi-square approximation. The computer program JMP Pro 16 was used for all analyses (SAS Institute Inc, Cary, NC, USA, 1989–2021).

## Results

### Effects of Light Treatment and Aphid Herbivory on CBD and THC Concentrations

Hemp with aphids had mean CBD concentrations of 3.96% ± 0.20 in bud tissues, compared with the control concentration of 5.16% ± 0.23 (Table 3; *F*_1,31_ = 16.09, *P* = 0.0005). Aphid herbivory also reduced THC bud concentration with a mean of 0.175% ± 0.010, lower than the 0.240 ± 0.010 in the control (Table 3; *F*_1,31_ = 23.18, *P* < 0.0001). Subsequently, aphid herbivory increased the CBD:THC ratio (Table 3; *F*_1,31_ = 8.05, *P* = 0.009). There were no significant effects of light quality on any of the plant chemistry measurements nor an interaction between light quality × herbivory (Table 3).

**Table 3.** The main effects of light treatment, aphid treatment, and their interactions were analyzed against the following *y*-variables using ANOVA: CBD concentration of bud tissue (% CBD), THC concentration in bud tissue (% THC), CBD:THC ratio of bud tissue (CBD:THC), bud mass per plant, leaf-stem mass per plant, the percentage of the plant’s above-ground mass that consisted of buds (% bud mass), plant height, the week when the plant’s apical cola reached 2.5 cm in length (cola development time), and the number of aphids counted on hemp leaflets at 13 wk (aphid density)

	Light treatment		Aphid treatment		Light × Aphid	
	*F* stat	*P*-value	*F* stat	*P*-value	*F* stat	*P*-value
% CBD	*F*_3,31_ = 1.39	0.271	*F*_1,31_ = 16.09	0.0005	*F*_3,31_ = 1.07	0.382
% THC	*F*_3,31_ = 1.67	0.200	*F*_1,31_ = 23.18	<0.0001	*F*_3,31_ = 1.67	0.398
CBD:THC	*F*_3,31_ = 0.41	0.744	*F*_1,31_ = 8.05	0.009	*F*_3,31_ = 0.23	0.231
Bud mass (g)	*F*_3,164_ = 2.68	0.048	*F*_1,164_ = 34.82	<0.0001	*F*_3,164_ = 11.86	0.784
Leaf-stem mass (g)^a^	*F*_3,169_ = 11.86	<0.0001	*F*_1,169_ = 40.26	<0.0001	*F*_3,169_ = 0.71	0.546
% Bud mass	*F*_3,153_ = 3.54	0.016	*F*_1,153_ = 61.13	<0.0001	*F*_3,153_ = 11.86	0.526
Height (cm)	*F*_3,167_ = 6.25	0.0005	*F*_1,167_ = 3.83	0.052	*F*_3,167_ = 1.59	0.194
Time to cola development	*F*_3,191_ = 78.18	<0.0001	*F*_1,191_ = 9.63	<0.0001	*F*_3,191_ = 19.18	<0.0001
Aphid density (aphids/leaflet)	*F*_3,47_ = 1.31	0.283	*F*_1,47_ = 51.80	<0.0001	*F*_3,47_ = 1.15	0.342

^a^Variable was log-transformed for statistical analyses.

### Effects of Light Treatment and Aphid Herbivory on Hemp Growth

**Fig. 1.**
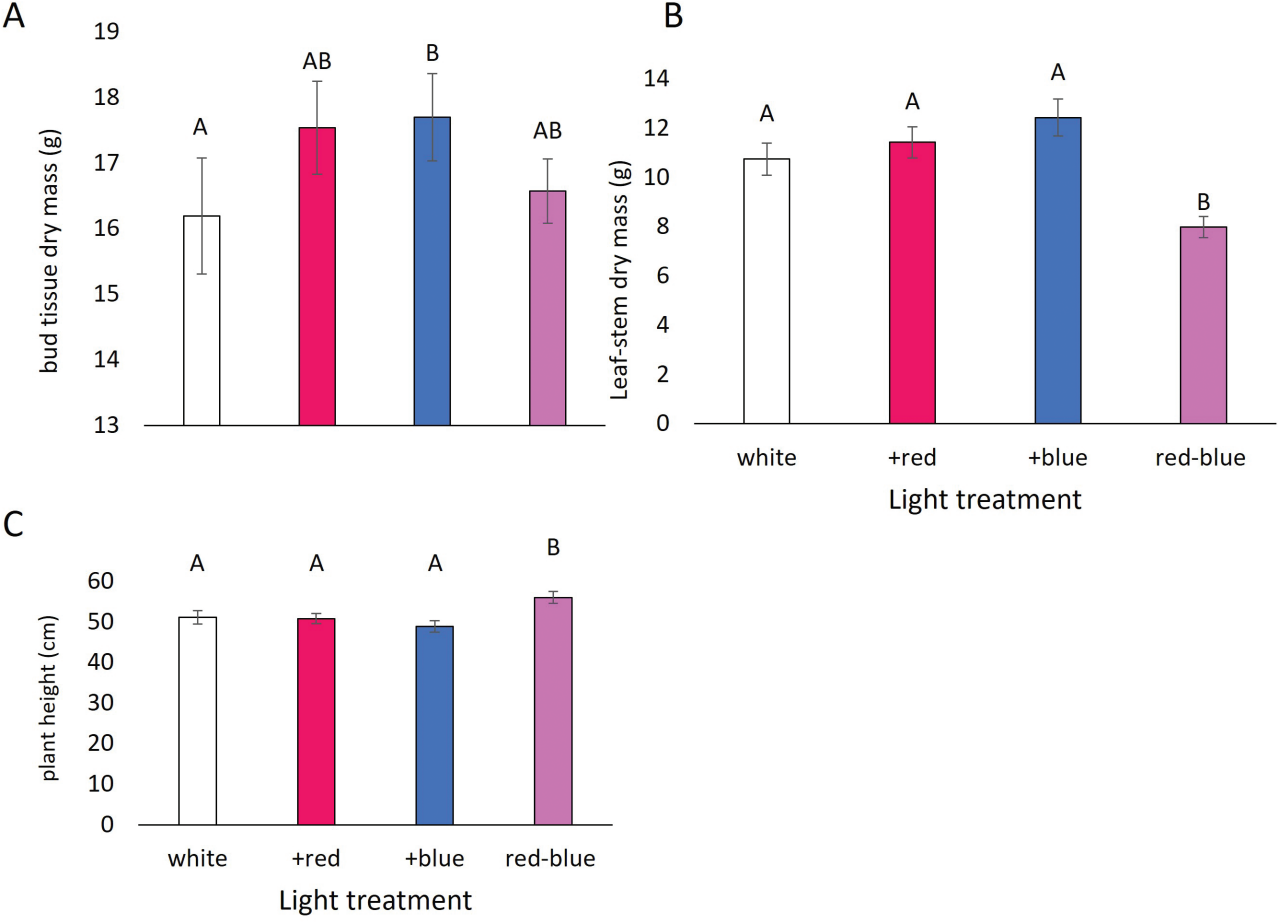
Light quality impacted the mean dry mass (g) of bud tissue (A), the mean dry mass (g) of leaf-stem tissue (B) and the mean height (cm) of hemp plants (C). Letters represent statistical significance between treatment groups, and those that do not have overlapping letters are different.

**Fig. 2.**
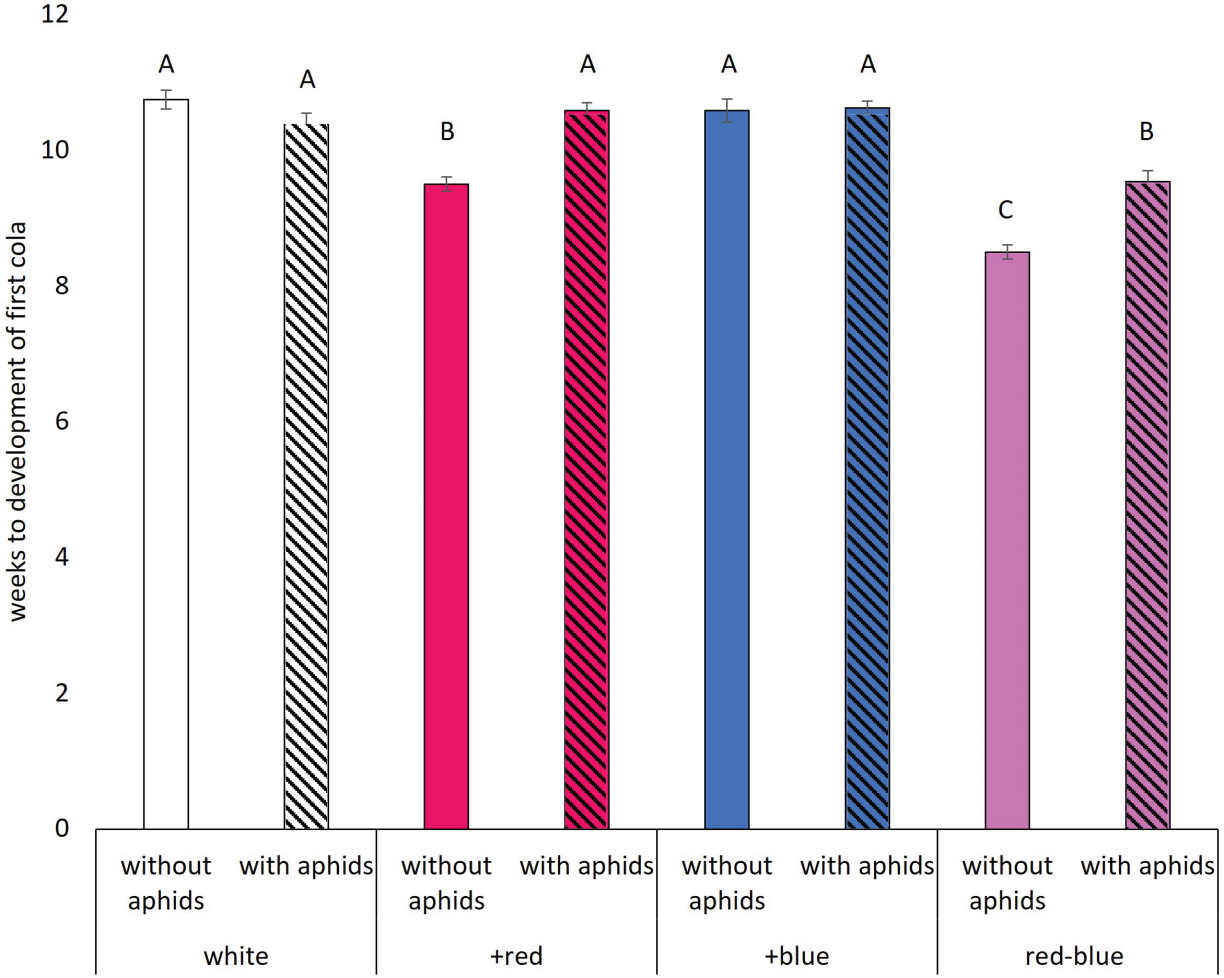
There was a significant interaction between light quality and herbivory during the time to the development of the first cola for hemp plants. Plants grown under white or +blue light were not impacted by the presence of herbivory. Plants grown under +red or red–blue light saw an increase in the time to the development of the first cola in the presence of aphid herbivores, and these times were different than the other light treatments. Letters represent statistical significance between treatment groups, and those that do not have overlapping letters are different.

Hemp infested with aphids yielded lower bud dry mass (15.16 ± 0.43) compared with the control (18.81g ± 0.49; Table 3; *F*_1,164_ = 34.82, *P* < 0.0001), a 19% decline. However, the aphid-treated plants had a higher leaf-stem dry mass (12.70 ± 0.53) than the control (8.99g ± 0.36; Table 3; *F*_1,169_ = 40.26, *P*  < 0.0001), a 41% increase. As for the effects of light treatment, the +blue treatment yielded 9% more bud dry mass than the control (Table 3; *F*_3,164_ = 2.68, *P* = 0.048; Fig. 1A). The red–blue treatment had a significantly lower leaf-stem dry mass than the other 3 light treatments (Table 3; *F*_3,169_ = 11.86, *P* < 0.0001; Fig. 1B), 25% less than the control white light and taller plants (Table 3; *F*_3,167_ = 6.25, *P* = 0.0005; Fig. 1C), 9% taller than the control. The time to the first cola development was the only metric where we saw an interaction between light and herbivory treatments (Table 3; *F*_3,191_ = 19.18, *P* < 0.0001; Fig. 2). Plants grown under the white and +blue light treatments took the longest time to develop the first cola on the plant, regardless of the presence or absence of aphid herbivores. Plants grown under the +red and red–blue light treatments developed the first cola sooner in the absence of herbivores, with the red–blue plants being the first plants to develop the first cola.

### Chamber Conditions

Three PPFD measurements were taken with a UPRtek (Zhunan Township, Taiwan) PG100N handheld spectrometer 5-cm above the growing medium in the center of the chamber per light treatment before the hemp was seeded. During the day cycle, the PPFD ranged from 118 to 123 μmol·m^−2^·s^−1^ (Table 2). During the 4-h night cycle, the +red treatment had a PPFD of 4.1 ± 0.1 μmol·m^−2^·s^−1^. All other remaining treatments had a PPFD of 0 μmol·m^−2^·s^−1^ during the night cycle. In both chambers, the average day temperature was 25.0 °C ± <0.1 and the average night temperature was 20.0 °C ± <0.1. The relative humidity measured in chamber 1 was 70.7% ± 0.1 (day) and 70.7 ± 0.2 (night). In chamber 2, day/night relative humidity was 76.4% ± 0.0 and 77.5% ± 0.1, respectively.

### Aphid Development

When examining the impacts of light quality on aphid life-history traits, there was no significant difference in time to the first molt, which occurred on day 3.8 ± 0.2 (Table 4; χ^2^_3, 41_** = **2.76, *P* =** **0.433). The day on which the aphids reached reproductive maturity (offspring first observed) occurred on day 9.2 ± 0.2 and did not differ between light treatments (Table 4; χ^2^_3, 33_** = **5.60, *P* =** **0.133).

**Table 4.** Means and standard deviation of aphid light history traits recorded under various light treatments. Values are rounded to 2 decimal places

Light treatment	Number of aphids observed	Days to first molt (mean ± standard error)	Number of aphids survived to reproductive maturity	Days to reproductive maturity (mean ± standard error)
White	7	4 ± 0.218	4	9.91 ± 0.285
+Red	14	3.79 ± 0.114	11	8.91 ± 0.415
+Blue	8	3.75 ± 0.232	8	9.33 ± 1.202
Red–blue	12	3.75 ± 0.463	11	8.5 ± 0.423
		χ^2^_[3, 41]_** = **2.76 *P* =** **0.43		χ^2^_[3, 33]_** = **5.60 *P* =** **0.13

### Aphid Fecundity

**Fig. 3.**
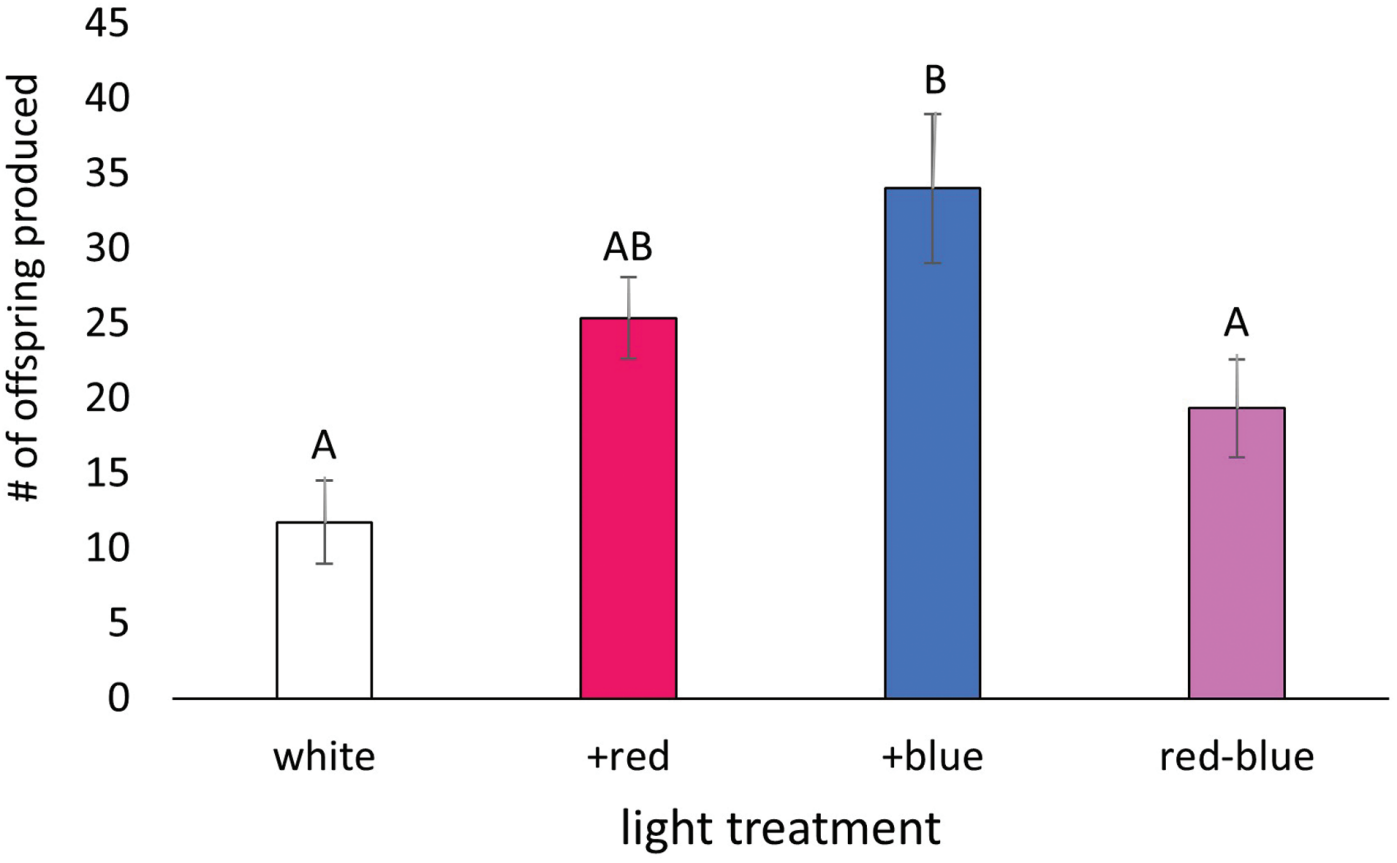
The effect of light treatment on the number of offspring individual aphids produced over 5 days. The 5-day period observed was days 2–6 of the aphid’s reproductive period. Letters represent statistical significance between treatment groups, and those that do not have overlapping letters are different.

Aphids in the +blue treatment produced the most offspring over the 5-day observation period, averaging 34.0 ± 5.0 offspring (Fig. 3), 189% more than the white control. The fecundity of the white and red–blue treatments was lower than the +blue treatment, producing an average of 11.8 ± 2.8 and 19.3 ± 3.2 offspring, respectively (*F*_3,29_ = 5.04, *P* = 0.007). The +red treatment did not differ from any other treatments, producing an average of 25.4 ± 2.7 offspring.

### Aphid Longevity

**Fig. 4.**
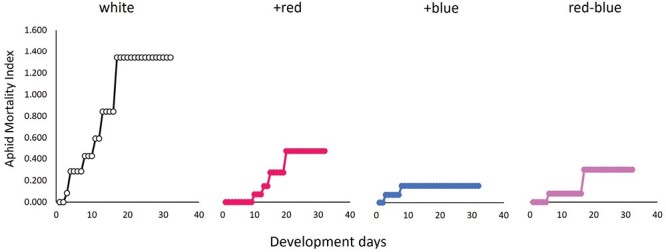
The aphid mortality index plotted over the developmental days of the aphid life-history observations. The dots represent the cumulative aphid mortality across time. The aphid mortality index is defined by the following formula: $y=\overset{32}{\underset{x=1}{\sum }}\left\{\frac{\;Natural\;\;\;deaths\;\;\;on\;\;\;Day(x)}{Aphids\;\;\;alive\;\;\;on\;\;\;Day(x)}\right\}+\left\{\frac{\;Natural\;\;\;deaths\;\;\;on\;\;\;Day(x+1)}{Aphids\;\;\;alive\;\;\;on\;\;\;Day(x+1)}\right\}$

The aphid mortality index indicated that aphids under the white light treatment suffered a significantly higher rate of natural mortality (χ^2^_3, 128_ = 42.32, *P* < 0.0001) compared with all other light treatments (Fig. 4).

## Discussion

This work was aimed at investigating the role of light quality as a management tool to manipulate plant–herbivore interactions using the indoor production of *C. sativa* L. and its primary pest herbivore, the cannabis aphid, *P. cannabis* as the model system. We aimed to answer the following questions: (i) How do light quality and herbivory interact to impact crop quality? (ii) Can light quality be used in an IPM plan to increase crop production?

### Plant Chemistry

In relation to manipulating plant chemistry, we found no direct effects of light quality on CBD or THC. The light treatments—white, +red, +blue, and red–blue—did not have any significant effect on CBD or THC concentration in hemp buds. The +blue treatment was predicted to have a higher CBD concentration because a high proportion of blue light can increase the expression of plant secondary metabolites (Livadariu et al. 2018, Magagnini et al. 2018, Eichhorn Bilodeau et al. 2019). This was not observed in our experiment and could be a result of the relative proportions of each light wavelength in our treatments. In our design, the +blue light treatment consisted of blue light representing 37% of the available light quality (Supplementary Fig. S1); in contrast, studies documenting the impacts of blue light on plant secondary chemicals have been performed with very restricted wavelengths (i.e., only blue; Livadariu et al. 2018) or under different daylength scenarios based on the type of *Cannabis* being grown (Magagnini et al. 2018). Our light treatments intentionally included a broader wavelength base to be more realistic of production conditions, and we conclude that such experiments need to be repeated to optimize the potential benefits of this tool for IPM.

Hemp plants that were infested with *P. cannabis* were predicted to have higher CBD and THC concentrations in their buds compared with control plants. This prediction was not supported and in fact hemp plants infested with *P. cannabis* had lower CBD and THC concentrations than those that did not experience herbivory. Despite our aphid infestations spreading to our control plants, this did not occur until after tissue was sampled to measure plant chemistry, and therefore, we are confident in this result. The decrease in CBD and THC observed in the aphid-infested plants may have been the result of resource reallocation. *Phorodon cannabis* feeds directly on the plant’s phloem. The main solute in phloem sap is sucrose, a photosynthate that is vital for fueling the plant’s metabolic processes, and amino acids essential for aphid nutrition (White 2012). Links between insect feeding, defensive mechanisms in plants, and the pathway to cannabinoid production in hemp lead to the common hypothesis that herbivory would lead to an increase in the production of cannabinoids (Sirikantaramas et al. 2005, Giordanengo et al. 2010, Jalali et al. 2019). Current literature shows that elevated levels of CBD reduce the performance of generalist chewing herbivores (Park et al. 2019, 2022, Abendroth et al. 2023). Piercing-sucking insects seem to decrease the concentration of CBD (Pulkoski and Burrack 2023), and at high levels, it can still be detrimental to their development (Hayes et al. 2023). *Cannabis* appears to be another interesting system to evaluate plant–insect–chemical defense interactions among various feeding guilds (chewing vs. piercing-sucking) and host ranges (generalist vs. specialist). Additionally, the implication for identifying economic thresholds and injury levels in the much-needed quest for IPM recommendations is a mighty task.

We can conclude, however, that cannabis aphids can influence the overall crop quality and management decisions need to consider the potential benefits of their infestation. If you are growing *Cannabis* for CBD extraction and THC is under strict regulation, herbivory may be a mechanism to decrease potential legal violations in THC content. In our work, a decline in THC concentrations from 0.23% to 0.17% after only 2 wk of aphid feeding is large enough to impact legal limitations, where a 0.01% change can move a crop into compliance. This decrease also comes at the cost of decreased CBD content, but the trade-off may be worth it if the crop can be salvaged. More research is needed to determine the plant age and herbivore density factors that contribute to the reduction in CBD and THC if herbivory is to be applied in the context of crop quality management.

### Plant Growth

The aphid-infested hemp had less dry mass in their buds, but more dry mass in their leaves and stems. The increased vegetative growth seen in the aphid treatment may have been an example of compensatory growth (McNaughton 1983, Smith 2005). This strategy can increase a plant’s ability to tolerate herbivory (Smith 2005). If the hemp plants were growing more vegetative tissue in response to the aphids, that may have reallocated resources away from bud growth and contributed to their lower mass.

The light treatments had varying effects on plant growth metrics. Hemp under the +blue yielded more bud dry mass than the white light treatment but did not differ significantly from the +red and red–blue treatments. It was unexpected that the white light treatment differed from the +blue because white is the most similar treatment regarding the PPFDs of red, blue, and green light (Table 1). The red–blue light treatment differed from all other treatments by having the lowest leaf-stem dry mass while achieving the tallest plant height and maintaining comparable bud yield. The red–blue treatment differed from the others by having a higher PFD of red wavelengths and an exceptionally low PFD of green wavelengths (Table 1). This is due to the red–blue LED arrays being comprised of red and blue diodes, while the LEDs used in the other treatments were primarily white, broad-spectrum diodes. Greenlight plays a role in regulating the growth of *Cannabis* leaves and stems (Lalge et al. 2017) and the red–blue treatment had the least amount of green compared with all other treatments (Supplementary Fig. S1). Because higher proportion of blue light can cause plants to grow shorter, the increased height observed in the red–blue treatment may have been the result of the red–blue treatment having the lowest proportion of blue light (Table 1, Supplementary Fig. S1).

The only plant metric that was influenced by the interaction between herbivory and light quality was the time to development of the first cola (Table 3). The presence of aphids increased the time to the first cola development among the +red and red–blue light treatments but did not impact cola development in the white or +blue treatments (Fig. 2). The development of the first cola or the onset of flowering sometimes is a stress-induced response. In hemp in particular, evidence of the role of light quality as the main driver for the onset of flowering is accumulating (Magagnini et al. 2018, Backer et al. 2019, Petit et al. 2020). Taken together, the red–blue light treatment, in the absence of aphids, was the first to flower, achieved the tallest plant height, and did not differ in bud yield compared with all other treatments. Red–blue light may be the most economical light spectrum to maximize yield while minimizing the allocation of resources to other plant tissues and the impacts of aphid herbivory on crop quality.

### Aphid Biology

To examine the impacts of light quality on the herbivore, we performed detailed life-history assays following individual aphids from birth to death under the 4 different light treatments. Because we cannot rear this insect in the absence of their host plant, there may still be some light quality-host plant interactions at play. However, we aimed to minimize these effects to the best of our ability through the standardization of temperature, day length, and plant genetics. Given these constraints, we saw no impact of light quality on the time to first molt and time to reproductive maturity for *P. cannabis*. However, light quality did influence the number of offspring produced with the +blue treatment producing more offspring than the white and red–blue light treatments and was antithetical to our prediction that it would yield the lowest fecundity. This original prediction was based on our hypothesis that the higher ratio of blue light would increase the plant’s production of secondary metabolites—including defensive compounds—and that the increase in allelopathic chemicals would be a detriment to the aphid.

There are many possibilities as to why this hypothesis—blue light harming aphids by upregulating host plant defenses—was not supported by the results of this experiment. Monophagous insect herbivores can be specially adapted at mitigating, or even utilizing, the defenses of their host plant (Ali and Agrawal 2012). Perhaps *P. cannabis* utilizes metabolites from the *Cannabis* defense response as nutrition, a hypothesis that would correlate higher fecundity observed under the +blue treatment, however we did not see a difference in CBD or THC content under the +blue treatment. There are a variety of other cannabinoids in the plant that this aphid specialist could be utilizing and were not measured in our study and have been implicated in herbivore performance on hemp (Hayes et al. 2023). In *Arabidopsis*, being treated with constant blue light induced the degradation of the plant’s defensive protein HRT, increasing the plant’s susceptibility to *Turnip crinkle virus* (Jeong, Chandra-Shekara, et al. 2010). There is a possibility that blue light—and other light wavelengths—could impact the stability of defensive compounds in *Cannabis*, reducing the plant’s ability to defend itself. Light quality can also trigger changes in host plant nutritional quality that benefit aphids (Dáder et al. 2014). Therefore, the differences in aphid fecundity observed may have been the result of differences in plant chemistry that was beyond the scope of the study.

Light quality also impacted early mortality in this experiment with the white treatment experiencing higher mortality at the onset of the experiment and consequently experiencing a higher mean on the aphid mortality index (Fig. 4). The white light treatment had similar light quality to the +red during the day cycle and sits between +blue and red–blue when comparing their proportions of red, green, and blue light (Supplementary Fig. S2). This experiment bears repeating to see whether the relationship between the white treatment and high natural mortality persists as well as integrating more biochemical analyses of the plant tissue to identify the potential mechanisms mediating the differences observed in early mortality and reproductive capacity.

### Conclusions

The main goals of pest management are to limit the amount of yield reduction that may be imposed by potential crop pests. In the context of CBD hemp production, we want to minimize the impact on overall yield (bud biomass) and maintain CBD:THC ratios to maximize content within the legal limits. The utility of herbivory and light quality as pest management tools has been documented in this work. In the absence of aphids, increased blue light leads to higher bud mass and CBD in the hemp variety ‘Little Giant’. In the presence of aphids, white light has the potential to reduce their initial growth and survival, but establishment will occur. In this situation, red–blue light may increase cola production and result in a similar quality yield compared to other light treatments. However, as is true for many management tools, the results are context dependent and come with several trade-offs. Implementing light quality or herbivory as a management tactic has a long way to go but remains a viable option to be explored. These results are promising and offer new tools for integrated pest management in controlled environments.

## Supplementary Material

nvad127_suppl_Supplementary_Material
