# Supplementary material for: Evaluating the impact of light quality on plant–herbivore interactions using hemp as the model system
Source: Environ Entomol. 2024 Jan 10;53(1):40–9. doi: 10.1093/ee/nvad127 (PMC10878358; doi:10.1093/ee/nvad127)

**Supplemental Tables and Figures**

**Supplemental Table S1.** The mean number of aphids on the experimental plants measured at two different time points after aphid infestations.

| Mean aphids per leaflet | white | +red | +blue | red-blue |
| --- | --- | --- | --- | --- |
| With aphids, week 10 | 85.1 ± 27.7 | 71.9 ± | 34.8 ± 12.9 | 291.3 ± 33.8 |
| Without aphids, week 13 | 34.3 ± 4.3 | 28.3 ± 4.6 | 25.5 ± 4.5 | 30.8 ± 2.9 |
| With aphids, week 13 | 418.5 ± 79.0 | 373.5 ± 135.1 | 200.8 ± 31.2 | 365.5 ± 64.2 |

**Supplemental Figure Legends**

**Supplemental Figure S1.** The timeline of hemp growth, aphid infestations, sampling and population management.

**Supplemental Figure S2.** Composition of each light color into the treatment for the plant-herbivore-light quality experiment. Each pie graph shows the percentage of red light (600-700 nm), green light (500-600 nm), and blue light (400-500 nm) in each light treatment’s PPFD (photosynthetic photon flux density). Both the day light quality and night light quality of the +red treatment is displayed because it was the only treatment to receive supplemental light at night. Values rounded to nearest whole number.

**Supplemental Figure S3.** Composition of each light color into the total treatment for the growth chamber experiment examining aphid life history traits. Each pie graph shows the percentage of red light (600-700 nm), green light (500-600 nm), and blue light (400-500 nm) in each light treatment’s PPFD (photosynthetic photon flux density). Both the day light quality and night light quality of the +red treatment is displayed because it was the only treatment to receive supplemental light at night. Values rounded to nearest whole number.

**Supplemental Figure S1.**
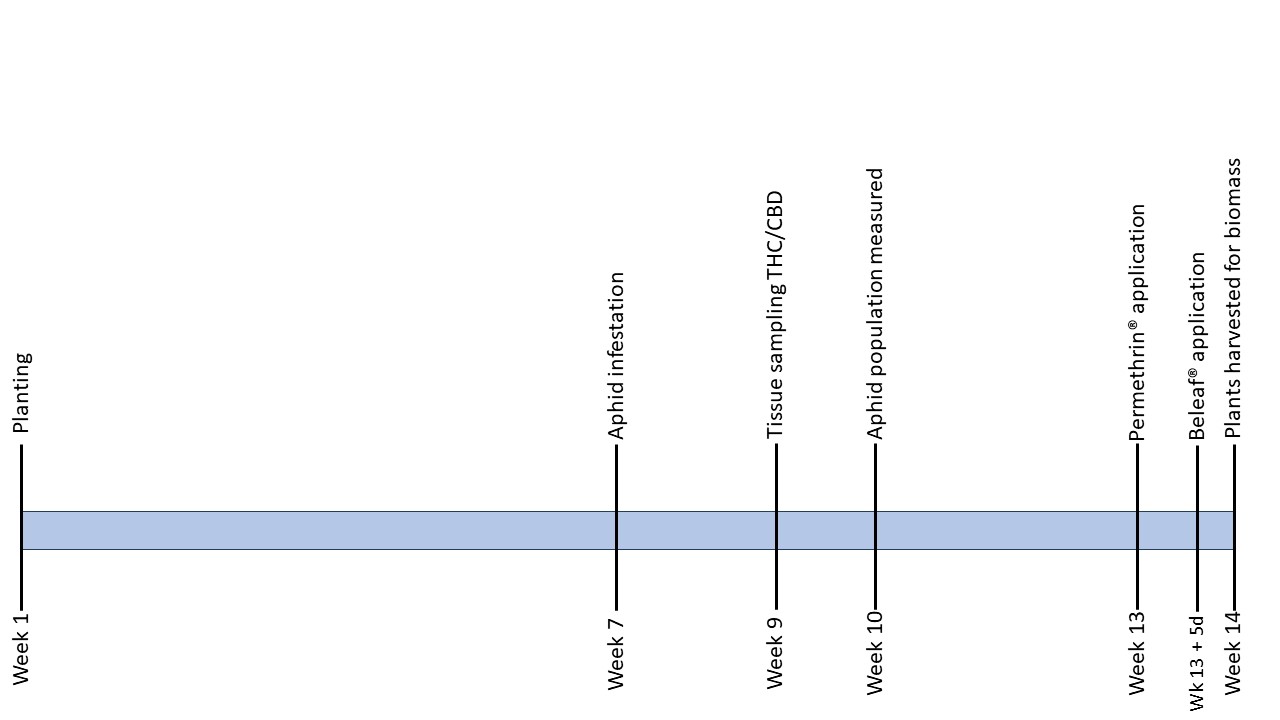


**Supplemental Figure S2.**
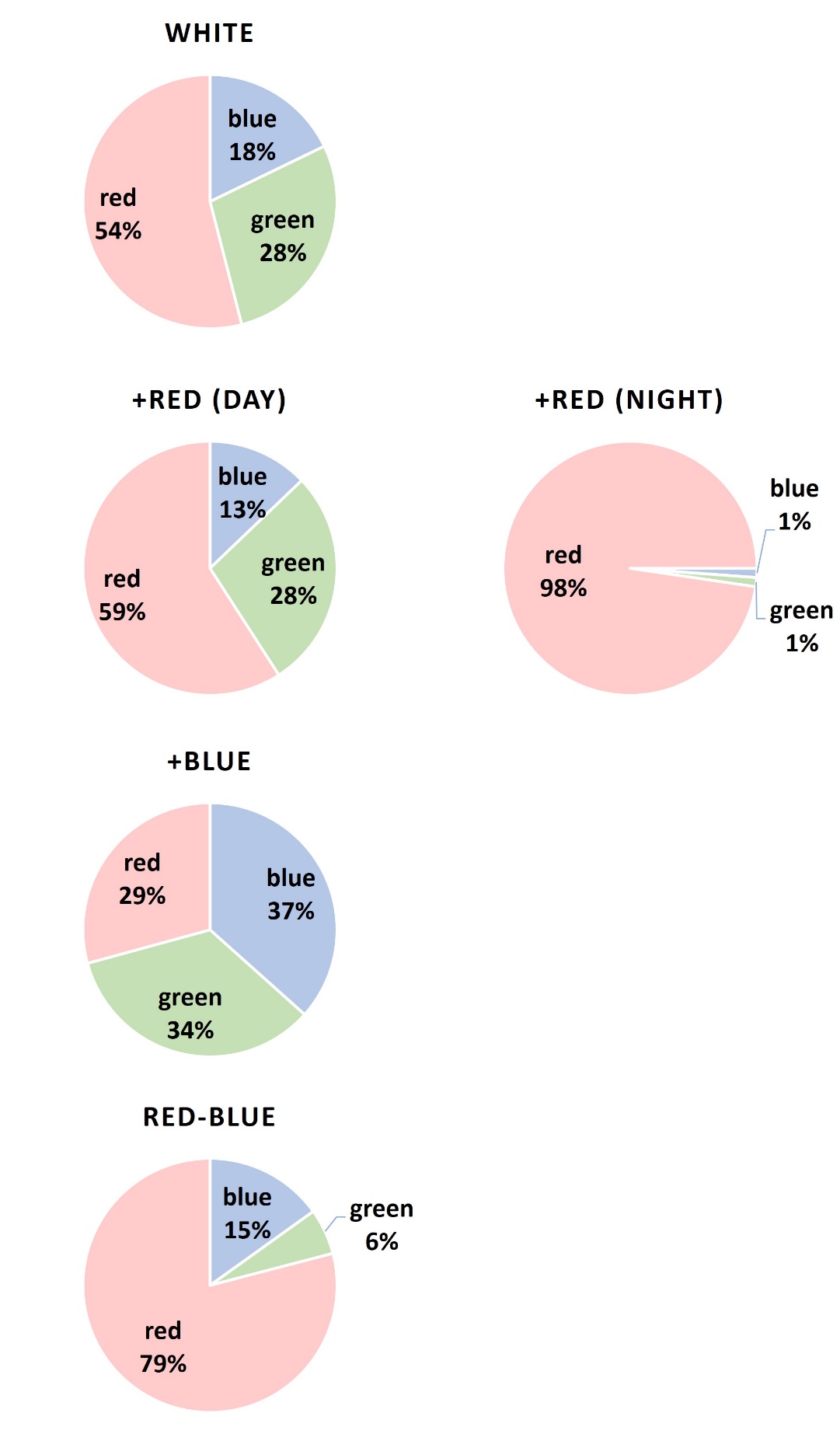


**Supplemental Figure S3.**
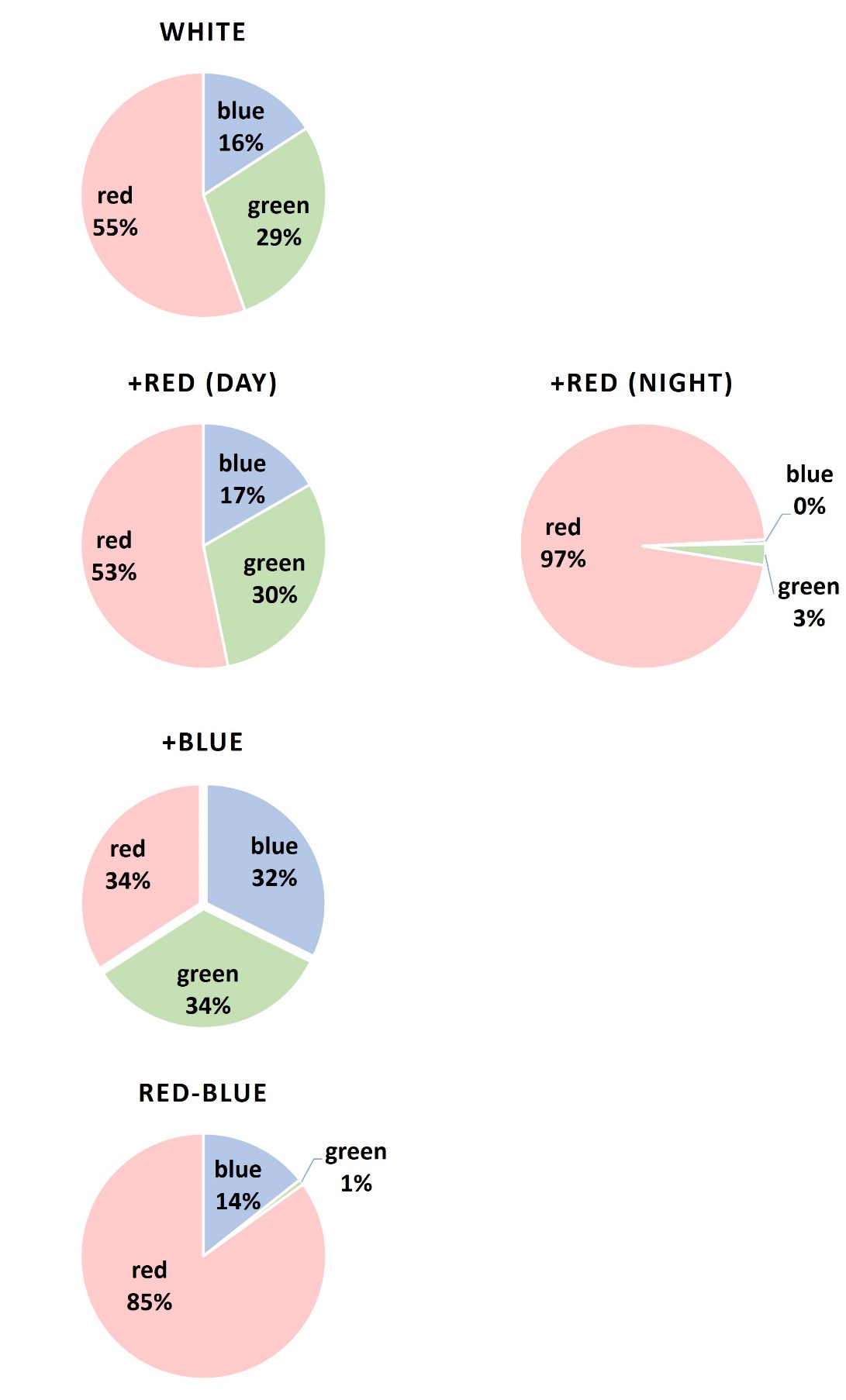

Supplement: nvad127_suppl_Supplementary_Material [file nvad127_suppl_supplementary_material.docx]
